# An Herbal Formulation of Jiawei Xiaoyao for the Treatment of Functional Dyspepsia: A Multicenter, Randomized, Placebo-Controlled, Clinical Trial

**DOI:** 10.14309/ctg.0000000000000241

**Published:** 2020-10-08

**Authors:** Guang Chen, Peimin Feng, Shaofeng Wang, Xiangping Ding, Jiaying Xiong, Jianhua Wu, Lihua Wang, Wei Chen, Guixia Chen, Mei Han, Ting Zou, Lei Li, Hongbo Du

**Affiliations:** 1 Division of Gastroenterology, Dongzhimen Hospital, Beijing University of Chinese Medicine, Beijing, China;; 2 Key Laboratory of Chinese Internal Medicine of Ministry of Education and Beijing, Dongzhimen Hospital, Beijing University of Chinese Medicine, Beijing, China;; 3 Division of Gastroenterology, Hospital of Chengdu University of Traditional Chinese Medicine, Chengdu, China;; 4 Division of Gastroenterology, Changsha People's Hospital, Changsha, China;; 5 Division of Gastroenterology, Gansu Second Provincial People's Hospital, Gansu, China;; 6 Division of Gastroenterology, Loudi Central Hospital, Hunan, China;; 7 Division of Gastroenterology, Hengyang Chinese Medicine Hospital, Hengyang, China;; 8 Division of Gastroenterology, Harrison International Peace Hospital, Hengshui, China;; 9 Division of Gastroenterology, Xiang Yang No. 1 People's Hospital, Xiangyang, China;; 10 Geriatric Department, Luoyang No. 1 Hospital of TCM, Luoyang, China;; 11 Center for Evidence-based Chinese Medicine, Beijing University of Chinese Medicine, Beijing, China;; 12 Technology Center for Drug Research and Evaluation, Chinese Association of Traditional Chinese Medicine, Beijing, China.

## Abstract

To investigate the efficacy and safety of an herbal formulation of Jiawei Xiaoyao (JX) on gastrointestinal symptoms in patients with functional dyspepsia (FD) who had previously rejected standard therapies of proton pump inhibitors, H2 blockers, or *Helicobacter pylori* eradication.

**METHODS:**

A total of 144 adult men and women with FD according to the Rome III criteria were recruited at 9 sites in China from August 2017 to April 2019. Participants were randomized to receive either a JX pill or placebo (12 g/d, 6 g twice a day) for 4 weeks. The primary end point was the change in the total Gastrointestinal Symptom Score (GIS) from baseline to week 4. The secondary end points included the scores on the Hamilton Depression Scale and the Hamilton Anxiety Scale. The safety outcomes included the results of the complete blood count, the liver function test, the renal function test, urinalysis, the fecal occult blood test, and an electrocardiogram.

**RESULTS:**

Data from 141 patients (JX pill, n = 70; placebo, n = 71) were statistically analyzed. The mean ± SD of the GIS for the JX pill group at baseline and 4 weeks was 9.3 ± 3.1 and 3.8 ± 3.0, respectively; the mean ± SD of the GIS for the placebo group at baseline and 4 weeks was 9.5 ± 3.4 and 5.3 ± 4.4, respectively (change from baseline to 4 weeks in the JX pill group vs change from baseline in the control group, −1.3 points; *P* = 0.013). The JX pill group showed greater improvement in both the Hamilton Depression Scale and Hamilton Anxiety Scale scores from baseline to 4 weeks than the placebo group, but the difference was not significant. The total number of adverse events was 30 in the JX pill group vs 20 in the placebo group (*P* = 0.240).

**DISCUSSION:**

The JX pill was superior to the placebo in terms of improving the GIS in patients with FD but did not significantly improve depression or anxiety symptoms. These findings suggest that the JX pill may have a positive effect on the resolution of gastrointestinal symptoms in patients with FD who are seeking alternative therapies.

## INTRODUCTION

Functional dyspepsia (FD) is a condition with symptoms centered in the upper abdomen, including a sensation of pain or burning in the epigastrium, early satiety (unable to finish a normal-sized meal), and fullness during or after a meal, in the absence of any organic, systemic, or metabolic disease that is likely to explain the symptoms (1). According to the Rome III criteria, FD is diagnosed if the patients manifest ≥1 of these symptoms for the past 3 months with onset at least 6 months before diagnosis (2). FD can be divided into 2 subtypes based on different pathophysiologies and etiologies: postprandial distress syndrome and epigastric pain syndrome (3). The global prevalence of FD is between 5% and 11% (4), with relatively higher prevalences (10%–40%) in Western countries and lower prevalences (5%–30%) in Asia (5). In particular, the prevalence of FD ranges from 11% to 17% among Japanese patients who have medical checkups and 45%–53% among Japanese patients who seek medical care because of upper gastrointestinal symptoms (6).

The pathogenesis of FD typically involves multiple factors, such as the disturbance of gastric accommodation, slow gastric emptying, and gastric hypersensitivity with distention of the stomach (7). FD has also been shown to be associated with *Helicobacter pylori* infection (8). Smoking, alcohol intake, and sleep disorders might interact with the symptoms of FD (9,10). Psychological distress, particularly anxiety, is surprisingly also related to FD and might even precede the onset of FD symptoms (11); patients with FD score higher than average on psychosocial measures (6). Currently, both the American College of Gastroenterology and the Canadian Association of Gastroenterology state that *H. pylori* eradication is necessary for infected patients, and those who are not infected or do not respond to treatment should receive a trial of proton pump inhibitors (PPIs), tricyclic antidepressants, and prokinetic therapy (in this order) (12). However, refractory FD still exists in patients who do not respond to the therapies mentioned above; these patients could seek psychological therapy, but it is a conditional recommendation with very-low-quality evidence (12).

Traditional herbal medicine has been listed as a second-line treatment in the guidelines of the Japanese Society of Gastroenterology, although with weak recommendations (6). The Jiawei Xiaoyao (JX) pill, which contains the same components as Gamisoyo-San in Japan (13), is an herbal medicine that is broadly used throughout China, Japan, and Korea to promote Qi and blood as well as to nourish the spleen based on the framework of traditional medicine theory; it also relieves symptoms such as epigastric discomfort (14). The 6-g JX pill is composed of the following raw herbs: *Paeonia lactiflora* Pall., 1.8 g; *Atractylodes macrocephala* Koidz., 1.8 g; *Mentha canadensis* L., 0.36 g; *Bupleurum falcatum* L., 1.8 g; *Angelica sinensis* (Oliv.) Diels, 1.8 g; *Thespesia populnea* (L.) Sol. ex Corrêa, 1.8 g; *Glycyrrhiza inflata* Batalin, 1.44 g; *Paeonia × suffruticosa* Andrews, 2.7 g; and *Gardenia jasminoides* J.Ellis, 2.7 g.

The China Food and Drug Administration has approved the JX pill as a complementary and alternative therapy in the treatment of diseases such as anxiety disorder (15) and psychological stress–induced insomnia (16). However, no randomized controlled trial has been conducted to evaluate the effects of the JX pill on the symptoms of FD. Therefore, this study aims to determine the efficacy and safety of the JX pill for the treatment of FD as an additional study for postmarketing reevaluation (see CONSORT 2010 Checklist, Supplementary Digital Content 1, http://links.lww.com/CTG/A407).

## METHODS

### Study design

This study was a multicenter, randomized, double-blinded, placebo-controlled, clinical trial of the JX pill in patients with FD. The study was designed and conducted in accordance with the World Medical Association Declaration of Helsinki. The study protocol was checked and approved by Dongzhimen Hospital Beijing University of Chinese Medicine Medical Ethics Committee (No. DZMEC-JG-2017-19) and registered in the Chinese Clinical Trial Registry, one of the World Health Organization International Clinical Trial Registry Platforms (No. ChiCTR1900021965) (17). Written informed consent was obtained from all the participants before their enrollment.

### Study population

Outpatients were considered eligible if they were aged 18–75 years, met the Rome III criteria for FD (the absence of organic dyspepsia was confirmed by both endoscope and abdomen ultrasound) (18), had ≥1 clinical symptom of FD according to the Gastrointestinal Symptom Score (GIS) (19), and had a Hamilton Depression Scale (HAMD) (20) score between 7 and 17, and previously rejected standard FD therapies including PPIs or H2 blockers.

Patients were excluded from this study if they had a history of peptic ulcer, hemorrhage, atrophic gastritis, erosive gastritis, cholecystitis, pancreatitis, abdominal surgery, malignant tumors, or indigestion caused by taking aspirin, nonsteroidal anti-inflammatory drugs, or any other drugs. Patients were also excluded if they had rated the first item on the HAMD (depression emotion) ≥3 or the third item (suicide) ≥2, had alanine aminotransferase (ALT) or aspartate aminotransferase (AST) levels ≥1.5 times higher than the normal upper limit, or had Cr levels higher than the normal upper limit. We also excluded women who had signs of menopause or had a positive pregnancy test; patients who routinely took antianxiety drugs, antidepressants, acid-suppressing drugs such as PPIs and H2 receptor blockers, gastrointestinal motility drugs, gastric fundus drugs of 5-hydroxytryptamine 1A agonists, or anti–*H*. *pylori* drugs in the past 2 weeks; those who were unable to tolerate the administration of the JX pill or were allergic to this drug; and those who had participated in other clinical trials involving oral drugs within the past 3 months.

### Randomization and intervention

This trial was conducted at 9 centers in mainland China. Eligible participants were randomly assigned into 2 groups to receive either the JX pill or a placebo in a 1:1 ratio using stratified block randomization, with the study sites serving as a stratification factor and a block length of 4 designed by Random Allocation Software v2.0.0 (M. Saghaei, Isfahan University of Medical Sciences, Isfahan, Iran). Participants, researchers, outcome assessors, and biostatisticians were blinded to the treatment. The researchers at the sites obtained the randomization number and corresponding drug code for each enrolled participant through the central randomization system managed by the clinical research organization of Beijing KangPaiXing Medicine Science and Technology Company. The drug codes were attached after the manufacturing and packaging of the JX pill and placebo by this clinical research organization, and each randomization number and group assignment was printed on paper and put in an opaque and sealed envelope and then stored in a locked cabinet at each site in case there was a need for emergency unblinding. The study pill and placebo were identical in size, shape, and flavor, and they were labeled with sequential randomization numbers. Both the JX pill and the matching placebo were manufactured by Beijing TongRenTang & Co. The dosage used in this trial was 6 g of JX pill or placebo 2 times daily, and the duration of the therapy was 4 weeks. Participants attended follow-up appointments at the second, fourth, sixth, and eighth week after treatment.

### Efficacy outcome measures

The primary outcome measure was the change in the total GIS from baseline to the end of the 4-week intervention. The GIS was recorded in a face-to-face interview where patients were asked by researchers about their symptoms and then rated the intensity of the symptoms on a 5-point Likert scale. The GIS is a well-validated measure of the overall severity of FD-associated gastrointestinal symptoms (19). The total score ranges from 0 to 40 points, with higher scores indicating more severe symptoms. Symptom severity was assessed by a 5-point Likert scale, and the response options were none (0), slight (1), moderate (2), severe (3), and very severe (4).

The second outcome in this study included GIS obtained every 2 weeks during the 8-week study period, the proportion of responders who were free of symptoms (defined as a GIS of 0), and the proportion of participants who manifested FD conditions after reporting symptom relief. Two independent study researchers who were trained psychologists assessed depression using the HAMD total score (scores range from 0 to 54, with higher scores indicating a greater degree of depression), and the digestive symptoms relative to depression were also assessed using the 12th item of the HAMD. The study researchers also assessed the anxiety of the participants using the Hamilton Anxiety Scale (HAMA) total score (scores range from 0 to 56, with higher scores indicating a greater degree of anxiety) (21).

### Safety outcome measures

Safety outcome measures included a complete blood count; liver function tests of ALT, AST, alkaline phosphatase, total serum bilirubin, and γ-glutamyl transpeptidase levels; a renal function test of creatinine (Cr) levels; blood urea nitrogen levels; urinalysis results; fecal occult blood test results; and electrocardiogram results. We monitored any adverse events throughout the entire study period using a standard adverse event case report form. This form included occurrence time, severity, duration, effective measures, and outcome.

### Sample size estimation

The null hypothesis was that the change from baseline to 4 weeks of the GIS in the JX pill group would not be superior to the change in the placebo group. Therefore, the formula of superiority hypothesis was used to calculate the sample size (22). In our pilot study, the mean difference between the change of 2 groups from baseline to 4 weeks was 1.7, with a combined variance of 10.2. We used the formula with these parameters and a 1:1 ratio of allocation to estimate that 60 participants in each group would be needed to provide 90% power to detect this difference at a 2-sided significance level of 5%. Assuming a dropout rate of 20%, the sample size of this trial was 144.

### Statistical analysis

Statistical analysis was completed by an independent statistician who was blinded to the allocation of participants. Analyses were based on the intention-to-treat principle. Normally distributed continuous variables were presented as the mean ± SD, and nonnormally distributed continuous variables were presented as the median (interquartile range). The differences between 2 groups were assessed using analysis of covariance for normally distributed continuous variables with similar SDs, the Wilcoxon rank-sum test for nonnormally distributed continuous variables, and the χ^2^ test for categorical variables. In addition, analysis of covariance with the general linear model was used to test the effects of the centers by setting the site as a covariate. A prespecified subgroup analysis of the subtypes of FD was conducted by adding an interaction factor of subtype × group into the general linear model. Safety outcomes were analyzed by χ^2^ tests, and adverse events were compared by the Poisson regression model, where the incidence of participants with adverse events was the independent variable, and the group was the dependent variable. Missing data were multiply imputed using a set of baseline, week 2, week 4, and week 6 outcomes under the missing-at-random assumption. A 2-tailed *P* value of less than 0.05 indicated statistical significance, and all analyses were conducted using the software package R (version 3.5.3, R&R of the Statistics Department of the University of Auckland, Auckland, New Zealand).

## RESULTS

### Patients

**Figure 1.**
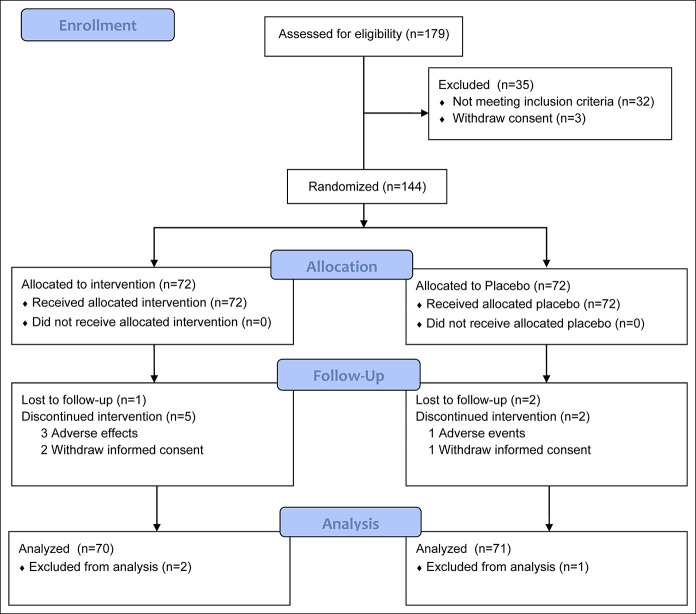
Flow diagram illustrating the number of participants in each group throughout the study.

From August 2017 to April 2019, 144 participants underwent randomization, and the flow diagram of participants throughout the study is presented in Figure 1. The trial ended in June 2019 when the last participant finished the follow-up, and the database was locked in July 2019. The rate of attendance during the 4-week intervention was 91.7% for the JX pill group and 94.4% for the control group. A total of 10 participants dropped out during the study: 6 in the JX pill group and 4 in the control group. Three participants were excluded from analysis due to consent withdrawal: 2 in the JX group and 1 in the control group. No significant differences were observed between the 2 groups in terms of the baseline characteristics before randomization (Table 1).

**Table 1.**
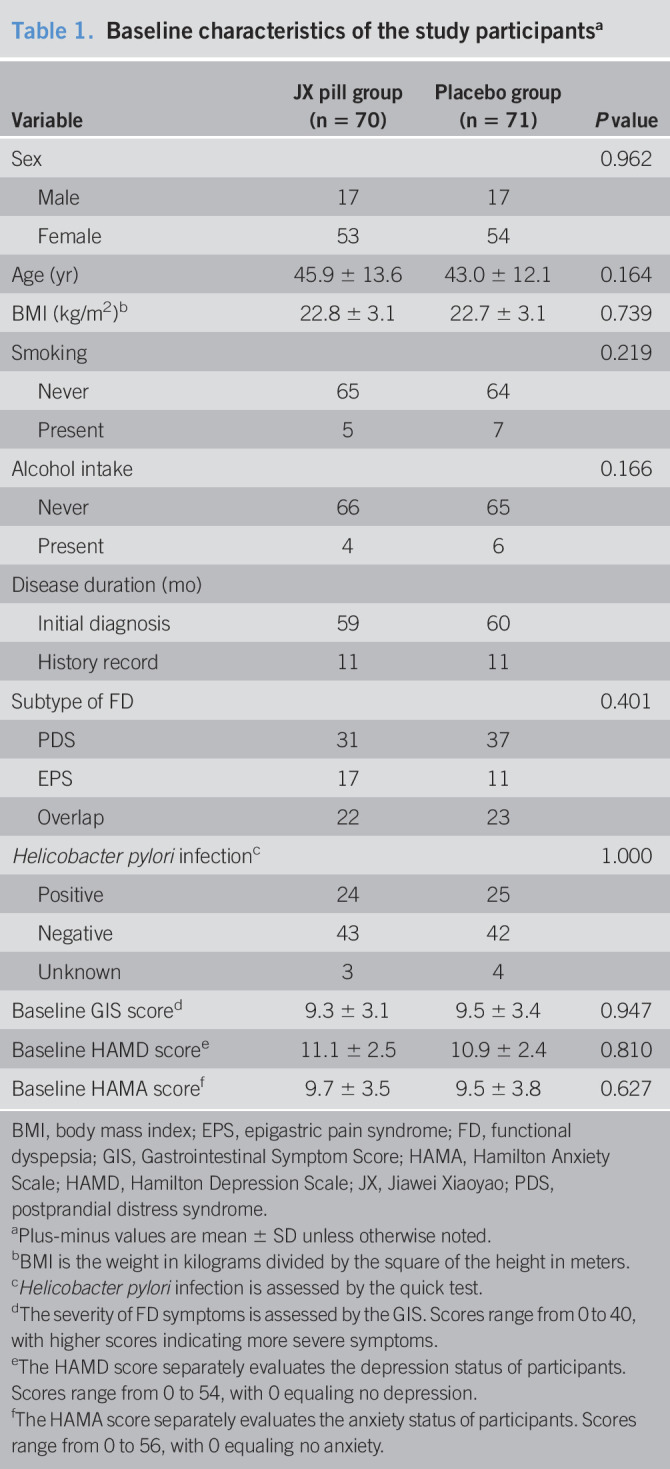
Baseline characteristics of the study participants^a^

Variable	JX pill group (n = 70)	Placebo group (n = 71)	*P* value
Sex			0.962
Male	17	17	
Female	53	54	
Age (yr)	45.9 ± 13.6	43.0 ± 12.1	0.164
BMI (kg/m^2^)^b^	22.8 ± 3.1	22.7 ± 3.1	0.739
Smoking			0.219
Never	65	64	
Present	5	7	
Alcohol intake			0.166
Never	66	65	
Present	4	6	
Disease duration (mo)			
Initial diagnosis	59	60	
History record	11	11	
Subtype of FD			0.401
PDS	31	37	
EPS	17	11	
Overlap	22	23	
*Helicobacter pylori* infection^c^			1.000
Positive	24	25	
Negative	43	42	
Unknown	3	4	
Baseline GIS score^d^	9.3 ± 3.1	9.5 ± 3.4	0.947
Baseline HAMD score^e^	11.1 ± 2.5	10.9 ± 2.4	0.810
Baseline HAMA score^f^	9.7 ± 3.5	9.5 ± 3.8	0.627

BMI, body mass index; EPS, epigastric pain syndrome; FD, functional dyspepsia; GIS, Gastrointestinal Symptom Score; HAMA, Hamilton Anxiety Scale; HAMD, Hamilton Depression Scale; JX, Jiawei Xiaoyao; PDS, postprandial distress syndrome.

a Plus-minus values are mean ± SD unless otherwise noted.

b BMI is the weight in kilograms divided by the square of the height in meters.

c *Helicobacter pylori* infection is assessed by the quick test.

d The severity of FD symptoms is assessed by the GIS. Scores range from 0 to 40, with higher scores indicating more severe symptoms.

e The HAMD score separately evaluates the depression status of participants. Scores range from 0 to 54, with 0 equaling no depression.

f The HAMA score separately evaluates the anxiety status of participants. Scores range from 0 to 56, with 0 equaling no anxiety.

### Response to treatment

**Figure 2.**
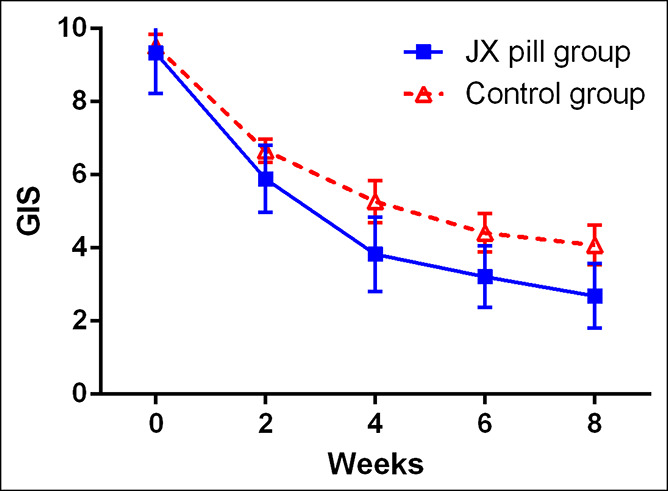
GIS during the 8-week intervention period. The GIS, measured at baseline, week 2, week 4, week 6, and week 8, is shown for the JX pill group and the control group. The GIS ranges from 1 to 40, with higher scores indicating more severe symptoms and lower scores indicating an improvement in outcomes. The values shown are unadjusted mean; I bars indicate 95% confidence intervals. GIS, Gastrointestinal Symptom Score; JX, Jiawei Xiaoyao.

Table 2 shows the changes in all outcomes from baseline to 4 and 8 weeks in the 2 groups. The mean (±SD) GIS at baseline and 4 weeks for the JX pill group was 9.3 ± 3.1 and 3.8 ± 3.0, respectively. The mean (±SD) GIS at baseline and 4 weeks for the placebo group was 9.5 ± 3.4 and 5.3 ± 4.4, respectively. At 4 weeks, the JX pill group had a significantly greater decrease in the GIS than did the control group (−5.5 points [95% confidence interval {CI}, −4.8 to −6.3] vs −4.2 points [95% CI, −3.5 to −4.9]). The mean difference between the 2 groups was −1.3 points (95% CI, −2.4 to −0.3; *P* = 0.013). Figure 2 shows that the mean between-group difference in the GIS gradually increased during the intervention; however, the overlap of the 95% CI between the 2 groups continued from baseline to week 8. Similarly, at 8 weeks, the JX pill group had a significant reduction in FD-related symptoms (change in the total GIS from baseline to 8 weeks, −6.7 points [95% CI, −5.9 to −7.4]), which was greater than the improvement in the control group; the mean between-group difference in the change from baseline to 8 weeks was −1.3 points (95% CI, −2.4 to −0.2; *P* = 0.016).

**Table 2.**
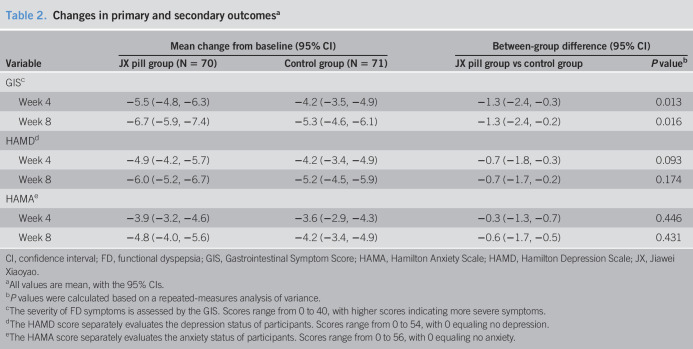
Changes in primary and secondary outcomes^a^

Variable	Mean change from baseline (95% CI)		Between-group difference (95% CI)	
	JX pill group (N = 70)	Control group (N = 71)	JX pill group vs control group	*P* value^b^
GIS^c^				
Week 4	−5.5 (−4.8, −6.3)	−4.2 (−3.5, −4.9)	−1.3 (−2.4, −0.3)	0.013
Week 8	−6.7 (−5.9, −7.4)	−5.3 (−4.6, −6.1)	−1.3 (−2.4, −0.2)	0.016
HAMD^d^				
Week 4	−4.9 (−4.2, −5.7)	−4.2 (−3.4, −4.9)	−0.7 (−1.8, −0.3)	0.093
Week 8	−6.0 (−5.2, −6.7)	−5.2 (−4.5, −5.9)	−0.7 (−1.7, −0.2)	0.174
HAMA^e^				
Week 4	−3.9 (−3.2, −4.6)	−3.6 (−2.9, −4.3)	−0.3 (−1.3, −0.7)	0.446
Week 8	−4.8 (−4.0, −5.6)	−4.2 (−3.4, −4.9)	−0.6 (−1.7, −0.5)	0.431

CI, confidence interval; FD, functional dyspepsia; GIS, Gastrointestinal Symptom Score; HAMA, Hamilton Anxiety Scale; HAMD, Hamilton Depression Scale; JX, Jiawei Xiaoyao.

a All values are mean, with the 95% CIs.

b *P* values were calculated based on a repeated-measures analysis of variance.

c The severity of FD symptoms is assessed by the GIS. Scores range from 0 to 40, with higher scores indicating more severe symptoms.

d The HAMD score separately evaluates the depression status of participants. Scores range from 0 to 54, with 0 equaling no depression.

e The HAMA score separately evaluates the anxiety status of participants. Scores range from 0 to 56, with 0 equaling no anxiety.

For the secondary outcome, the number of patients free of symptoms at week 4 was 50 (71.4%) in the treatment group vs 41 (57.8%) in the placebo group, indicating a potential difference (*P* = 0.064) between the 2 groups. Three patients in the treatment group experienced relapse during the 4-week follow-up, whereas 6 patients in the control group experienced relapse. The JX pill group had a greater improvement in the HAMD total score from baseline to 4 weeks than the placebo group, but the difference was not significant (mean between-group difference, −0.7 points [95% CI, −1.8 to −0.3]; *P* = 0.093). The JX pill group also had a greater improvement in the HAMA total score from baseline to 4 weeks than the placebo group, but the difference was not significant (mean between-group difference, −0.3 points [95% CI, −1.3 to −0.7]; *P* = 0.446).

**Figure 3.**
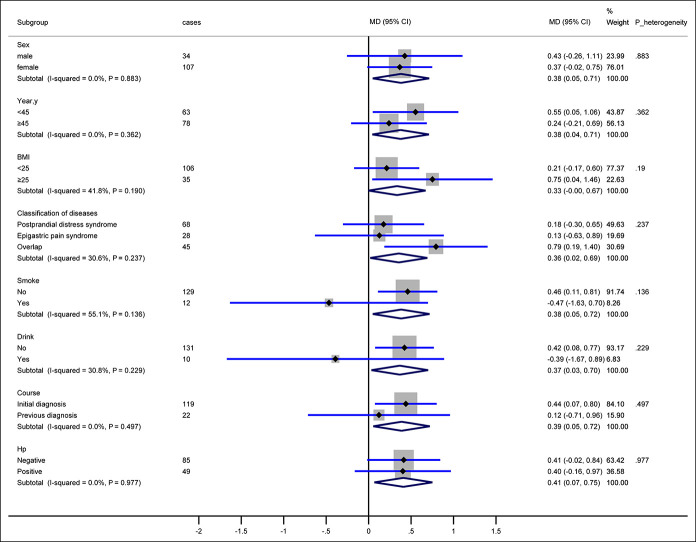
Changes from baseline to 4 weeks in the GIS by subgroups. The size of the data markers indicates the size of the standard mean difference of the GIS from baseline to week 4. *P* values were calculated by interaction parameters in the general linear model. BMI, body mass index; CI, confidence intervals; GIS, Gastrointestinal Symptom Score; Hp, *H.pylori*; MD, mean difference.

Figure 3 shows the results from the exploratory subgroup analyses in terms of sex, year, body mass index, FD subtypes, disease course, and *H. pylori* infection for the primary end points at week 4. The findings from subgroups were consistent with those obtained from the entire study population. However, differences potentially existed between the smoking subgroup (8.5% of the overall population) and the nonsmoking subgroup (91.5% of the overall population): −0.5 points (95% CI, −1.6 to 0.7) vs 0.5 points (95% CI, 0.1–0.8). There were also potential differences between the alcohol intake (7.1% of the overall population) and no alcohol intake subgroups (92.9% of the overall population): −0.4 points (95% CI, −1.7 to 0.9) vs 0.4 points (95% CI, 0.1–0.8).

### Safety assessment

A total of 141 participants were included in the safety outcome analyses (Table 3). The total number of adverse events was 30 in the JX pill group vs 20 in the placebo group (*P* = 0.240). Some participants reported more than 1 adverse event. The only severe adverse event in the JX pill group was the initial diagnosis of an ocular tumor, which was not related to the treatment. For the drug-related adverse events in the JX pill group, 1 participant reported slight diarrhea at day 2 after the treatment; 1 participant reported mild constipation at day 3; and 1 participant had abnormal liver function (ALT, 108.7 U/L; AST, 63.7 U/L) at week 2. The Poisson regression analysis of drug-induced adverse events and withdrawal revealed no difference between the 2 groups.

**Table 3.**
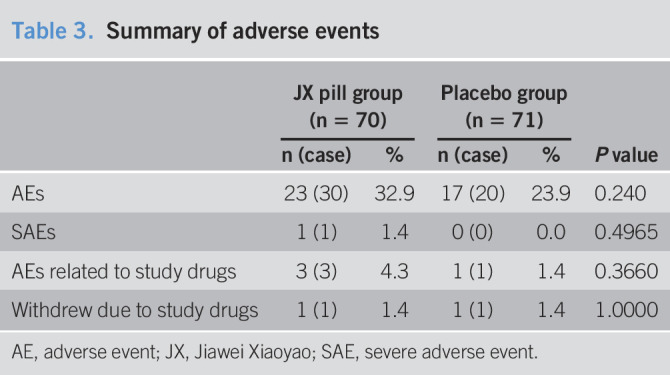
Summary of adverse events

	JX pill group (n = 70)		Placebo group (n = 71)		*P* value
	n (case)	%	n (case)	%	
AEs	23 (30)	32.9	17 (20)	23.9	0.240
SAEs	1 (1)	1.4	0 (0)	0.0	0.4965
AEs related to study drugs	3 (3)	4.3	1 (1)	1.4	0.3660
Withdrew due to study drugs	1 (1)	1.4	1 (1)	1.4	1.0000

AE, adverse event; JX, Jiawei Xiaoyao; SAE, severe adverse event.

## DISCUSSION

To our knowledge, this is the first registered randomized controlled clinical trial to investigate the efficacy of the JX pill compared with the placebo using GIS evaluation. We found that compared with the placebo, the JX pill seems to be effective in reducing the GIS during a 4-week intervention for patients with FD as diagnosed by the Rome III criteria and who had previously rejected standard therapies of PPIs, H2 blockers, or *H. pylori* eradication. Although the difference between the 2 groups in terms of the number of patients who were free of symptoms at week 4 was not statistically significant, the JX pill group had a significantly greater decrease in the GIS than did the placebo group, and this improvement was maintained at 8 weeks. Adverse effects should be monitored in the clinical application of the JX pill, especially regarding bowel movement disorders such as diarrhea, constipation, and abnormal liver function (ALT and AST levels).

Subgroup analysis indicates that the between-group difference in the GIS potentially differs between the smoking and nonsmoking subgroups and between the alcohol intake and no alcohol intake subgroups. The gastrointestinal impairment caused by nicotine and alcohol might partly explain these results (23,24); nicotine interacts with the central nervous system (25), which might lead to a change in the sensitivity of the potential targets of the JX pill. Previous studies have shown that smoking could aggravate FD-associated symptoms and that alcohol intake is also related to the occurrence of FD symptoms in Japan (9,10). Furthermore, the pharmacokinetic parameters from previous studies show that the oral administration of JX San, another sort of preparation with the same components as the JX pill, could be affected by puerarin in patients with FD (26). We assume that alcohol and smoking might also exert potent effects on the pharmacokinetic profile of the JX pill. Further refined strategies for patients with FD who smoke and consume alcohol deserve more mechanistic studies and clinical trials. Interestingly, subgroup analysis shows that no statistically significant difference existed in the efficacy of the JX pill between patients with *H. pylori* positivity and negativity, indicating that the JX pill might improve FD symptoms in both patients with *H. pylori* infection and patients without infection.

A previous study showed that Gamisoyo-San, a kind of medicine widely used in both Japan and Korea with the same herbal components as the JX pill, did not improve the anxiety of patients with generalized anxiety disorder; however, it was useful in reducing depressive symptoms among patients with anxiety disorder (15). Similarly, this trial shows that there was no significant improvement in HAMD and HAMA scores after a 4-week intervention by the JX pill among patients with FD.

The biological mechanisms by which the JX pill affects the clinical course of FD remain unclear. As a complex, multicomponent herbal medicine, the JX pill might perform diverse functions to relieve FD symptoms in terms of gastrointestinal motility. Previous animal studies of Kamishoyosan, a type of Kampo medicine with the same herbal material as the JX pill, demonstrated that the gamma-aminobutyric acid–benzodiazepine receptor complex is associated with the anxiolytic effect of Kamishoyosan in rats with a socially isolated ovariectomized model (27) and that it could ameliorate sociability deficits via dopamine D_1_ and gamma-aminobutyric acid receptor functions in ovariectomized mice (28). Moreover, Kamishoyosan could also improve the condition of autism spectrum disorder mice induced by a selective type I 5α-reductase inhibitor by facilitating a dopamine receptor–mediated mechanism (29). We hypothesize that the JX pill improves the symptoms of FD by regulating the brain-gut axis.

The participants in this study were relatively young, their body mass index was largely within a normal scale, and they mostly had very mild symptoms. Some of the participants were admitted to hospitals for yearly health examination; others participated in this trial because of the severity of the FD symptoms. Because of this feature of the enrolled patients, we could not detect a difference in the improvement of FD symptoms in patients with severe FD and with an overweight body. Current guidelines suggest *H. pylori* eradication is necessary for those who are infected, and a regimen of PPIs, tricyclic antidepressant, and prokinetic therapy should be suggested; however, the dyspepsia subgroup analysis in a meta-analysis of 6 trials showed that PPI therapy is less effective in the epigastric pain subgroup, with no statistically significant difference between the treatment and placebo groups in the dysmotility subgroup (30). There is no other option for patients who do not respond to the standard therapy. For herbal medicine broadly used in Asian countries, the European Society of Gastrointestinal Endoscopy also recommends in the guidelines the use of Moluodan, a preparation of Chinese herbal medicine, for the treatment of dysplasia in epithelial precancerous conditions and lesions in the stomach (31). The JX pill, which is also a Chinese medicine, could be an option to relieve the symptoms of FD and is expected to be recommended in the guidelines of FD treatment in the future when international trials confirm further evidence with a larger population.

Our study has limitations. First, the potential for selection bias might limit the generalizability of this trial because the patient populations include a group of patients with *H. pylori* positivity who rejected standard therapy (*H. pylori* eradication treatment, PPIs and H2 receptor blockers) and turned to alternative therapies, although they had been fully educated on the risks of failing to eradicate *H. pylori*. Second, gastrointestinal physiology tests, such as scintigraphy-based solid-phase gastric emptying studies, nutrient drink tests, and single-photon emission computed tomography imaging, were not used to investigate the efficacy of the JX pill in this study. Instead, we used the GIS to evaluate the efficacy of the JX pill in reducing FD symptoms, and a pilot study validated the value of the GIS. Third, daily symptom diaries assessing FD symptoms were not provided as an outcome to evaluate daily symptoms. However, self-reports of adequate relief between baseline and week 4 were analyzed to detect the meaningful clinical advantage of the JX pill compared with the placebo.

In conclusion, the JX pill appeared to relieve the symptoms of FD that was diagnosed using the Rome III criteria regardless of the subtype of postprandial distress syndrome or epigastric pain syndrome. Although adverse events were reported, there was no overall difference in the number of adverse events between the 2 arms. These findings suggest that at normal doses, the JX pill is relatively safe, but adverse effects such as abnormal liver function should be monitored. The results support the use of the JX pill in patients with FD who are seeking alternative therapies.

## CONFLICTS OF INTEREST

**Guarantor of the article:** Hongbo Du, MD, PhD.

**Specific author contributions:** H.D. planned the study and approved the final manuscript. G.C. interpreted the data, drafted the manuscript, and approved the final version of the draft. P.F., S.W., X.D., J.X., J.W., L.W., W.C., and G.C. conducted the study, collected the data, and approved the final manuscript. M.H., T.Z., and L.L. interpreted the data and approved the final manuscript.

**Financial support:** The study was funded by the China Association of Traditional Chinese Medicine (No. GZYPX2016032) and the BUCM Cultivation plan for a famous doctor (No.2018-98). The manufacturer has not been involved in the design, conduct, and interpretation of this trial.

**Potential competing interests:** None to report.

**Study Highlights**

**WHAT IS KNOWN**

✓ FD is a disorder in the gastroenterology system for which there is no available therapy approved by the FDA.
✓ The JX pill is an herbal medicine that has shown efficacy in relieving some sorts of symptoms of the gastroenterology system.

**WHAT IS NEW HERE**

✓ The JX pill, herbal medicine, was shown to relieve symptoms of FD after a 14-day intervention.
✓ The JX pill was found to be safe in the treatment of FD.

**TRANSLATIONAL IMPACT**

✓ The JX pill could be a potential treatment for patients with FD, and the results could change the current guidelines.
